# Necroptosis-mediated HMGB1 secretion of keratinocytes as a key step for inflammation development in contact hypersensitivity

**DOI:** 10.1038/s41420-022-01228-6

**Published:** 2022-11-07

**Authors:** Ni Lian, Yujie Chen, Sihan Chen, Ta Xiao, Changjun Song, Yangying Ke, Xuecui Wei, Chunyan Gong, Hui Yu, Heng Gu, Qing Chen, Min Li, Xu Chen

**Affiliations:** 1 Jiangsu Key Laboratory of Molecular Biology for Skin Diseases and STIs, Institute of Dermatology, Chinese Academy of Medical Sciences & Peking Union Medical College, Nanjing, 210042 China; 2 Key Laboratory of Basic and Translational Research on Immune-Mediated Skin diseases, Institute of Dermatology, Chinese Academy of Medical Sciences & Peking Union Medical College, Nanjing, 210042 China; 3 Center for Global Health, School of Public Health, Nanjing Medical University, Nanjing, Jiangsu 211166 China; 4 Department of Transfusion Medicine, Nanjing Drum Tower Hospital, The Affiliated Hospital of Nanjing University Medical School, Nanjing, Jiangsu 210008 China

**Keywords:** Immunology, Cell biology

## Abstract

Keratinocyte necroptosis (with proinflammatory characteristic) is required for epidermal damage in contact hypersensitivity (CHS). In DNCB-induced CHS mice model, we observed the aggravated keratinocyte death and increased phosphorylation level of MLKL, RIPK3 and RIPK1. However, CHS skin lesion did not present in keratinocyte-specific *Mlkl* knockout mice. We validated that MLKL-mediated keratinocyte necroptosis is required for epidermal damage in response to immune microenvironment in CHS. Moreover, MLKL-mediated necroptosis deficiency or inhibition resulted in blocking recruitment and activation of inflammatory cells in CHS via reducing HMGB1 release in keratinocytes. This study suggests that MLKL-mediated keratinocyte necroptosis functions as a self-amplified actor in inflammatory responses and could be considered as an effective therapeutic target. It proposes an innovative prospective that inhibiting keratinocyte necroptosis can prevent the development of epidermal damage in CHS.

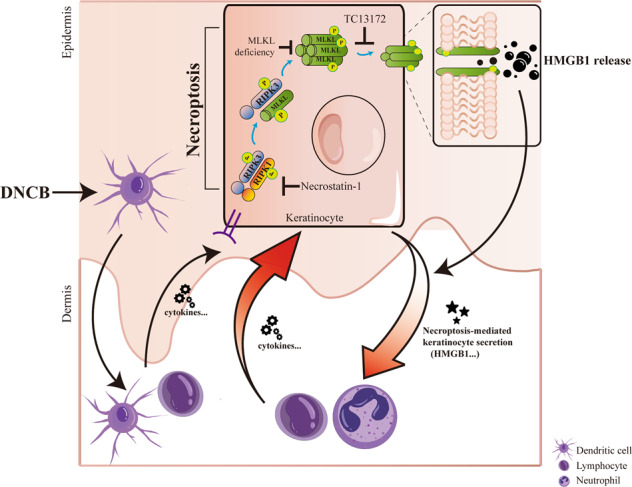

## Introduction

Contact hypersensitivity (CHS), also known as allergic contact dermatitis (ACD), is one common inflammatory skin diseases [1]. The incidence of ACD is increasing and is now the second leading cause of work-related illness in the United States, with a prevalence of 18%, affecting at least 10.6% of the working population [2, 3]. The typical histologic features of CHS present epidermal hyperplasia, epidermal spongiosis and dermal infiltration of inflammatory cells [4]. Activated T cells induce Factor associated suicide (Fas)-dependent keratinocyte apoptosis in skin tissue of ACD with T cells infiltration [5]. Keratinocyte apoptosis was reported to be in a high level in acute dermatitis of ACD. Activation of keratinocyte apoptosis by skin-infiltrating T cells leads to E-cadherin cleavage, and damage of demsosomal cadherins contributes to spongiosis formation in skin lesion of ACD [6]. Therefore, compared with infiltration of T cells, epidermal pathologic changes were considered as a more reliable indicator for evaluating eczematous severity for ACD [7]. Necroptosis is induced by challenges of intracellular or extracellular homeostasis and critically depended on Mixed lineage kinase domain-like protein (MLKL), Receptor interacting serine/threonine kinase 3(RIPK3), and RIPK1 [8, 9]. Emerging evidences indicate that necroptosis could be involved in pathogenesis of many inflammatory disorders [9]. However, the role of keratinocyte necroptosis in CHS remains unknown.

High mobility group box 1 (HMGB1) has been implicated as a pro-inflammatory protein in the pathogenesis of various inflammatory and autoimmune diseases, previous studies have identified the relevance of HMGB1 to inflammatory skin diseases [10–12]. Nevertheless, the contribution of HMGB1 secreted from keratinocytes to inflammatory skin diseases has not been well characterized.

Considering the renewed understanding of regulated cell death, we explored whether keratinocyte necroptosis plays a crucial role in the pathogenesis and development of inflammatory microenvironment, and HMGB1 secretion from necroptotic keratinocytes is involved in the regulation. And we explored whether inhibiting keratinocyte necroptosis is an effective therapeutic strategy for CHS.

## Results

### Necroptosis core regulators are activated in keratinocytes of CHS murine model

**Fig. 1:**
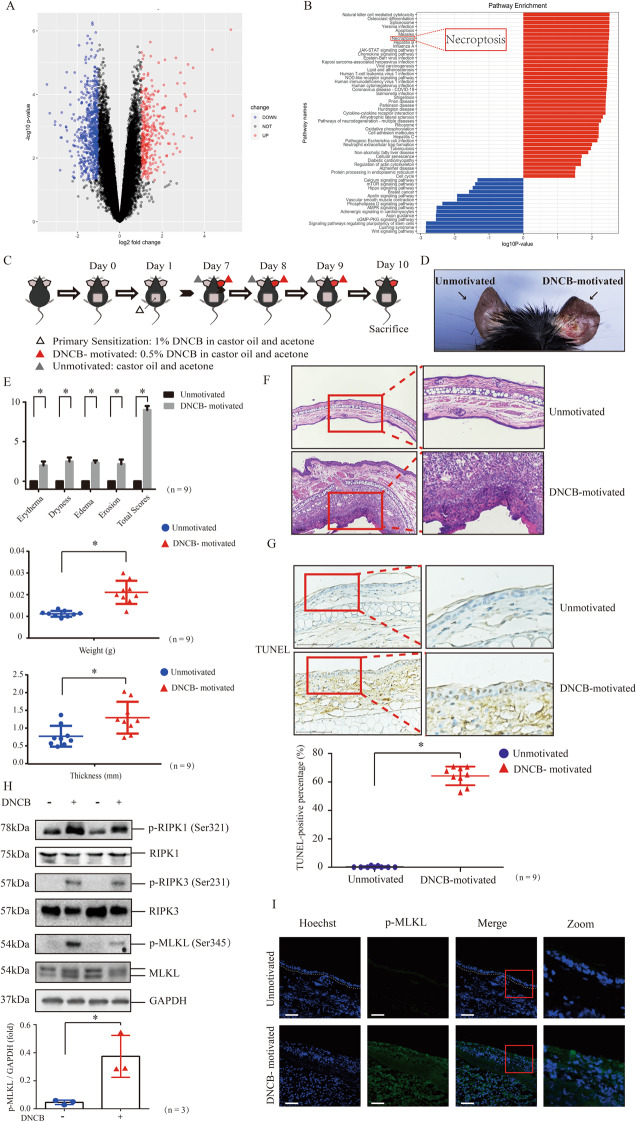
Necroptosis core modulators are activated in keratinocytes in CHS murine model. **A** Volcano plot displaying differential expressed genes between skin lesions and non-lesions of ACD patients based on Pedersen MB et. al’s study [13]. **B** KEGG pathway analysis of the differential expressed genes. Annotation clusters are shown according to their enrichment scores [log10 (*p*-value)]. **C** Flowchart of CHS murine model. **D** The skin appearance of DNCB-motivated CHS model. Left ear was the sample of unmotivated tissue, and right ear was motivated by DNCB. **E** The severity of skin manifestation in the CHS mice was evaluated by Dermatitis Score, ear weight and thickness. (*n* = 9). **F** The histological features were showed by the tissue section staining with Hematoxylin and Eosin (H&E, 20× magnification). **G** The level of keratinocyte death was determined by TUNEL staining. (*n* = 9). Scale bar represents 100 μm. **H** The protein level and phosphorylation of RIPK1, RIPK3 and MLKL were detected by western blotting. GAPDH served as the loading control. Statistical analysis of the interested protein was shown (*n* = 3). **I** The phosphorylation of MLKL was detected by immunofluorescence in the tissue of CHS mice model (green) (*n* = 9). Scale bar represents 100 μm. ∗*p* < 0.05.

The GSE database (accession number GSE6281) was mined using a dataset derived from skin biopsy samples of skin lesions or non-lesions of ACD patients from Pedersen MB et. al’s study [13]. We re-analyzed the differentially expressed genes in the 96h-motivated time set (Fig. 1A) based on the data base [13] and found significant enrichment on necroptosis pathway (Fig. 1B).

We established DNCB-induced CHS murine model in C57BL/6 J mice (Fig. 1C) [14, 15]. The DNCB-motivated ears presented typical manifestation of dermatitis (Fig. 1D-E). Scores for evaluating skin dermatitis included erythema, dryness, edema and erosion, and scores of all these items were increased in DNCB-motivated ears (Fig. 1E). Weight and thickness of DNCB-motivated ears were increased due to skin inflammation, compared with unmotivated ears (Fig. 1E). Histologically, DNCB-motivated ears showed that hypertrophy, edema and local epidermal necrosis and infiltration of inflammatory cells (Fig. 1F). Next, we determined the total level of keratinocyte death in dermatitis lesion using TUNEL staining in tissue sections. The data showed that keratinocyte death was increased in dermatitis lesion (Fig. 1G). Apoptosis was reported to occur in keratinocytes of skin lesion [5, 16, 17]. Interestingly, we found that phosphorylation levels of necroptosis core regulators, MLKL, RIPK3 and RIPK1 were increased in DNCB-motivated ears (Fig. 1H). We further validated that MLKL phosphorylation was increased in the DNCB-motivated ears (Fig. 1I). These data demonstrate that necroptosis core regulators are activated in skin inflammation during DNCB-induced contact hypersensitivity.

### DNCB-motivated CHS does not present in the keratinocyte-specific Mlkl cKO mice

**Fig. 2:**
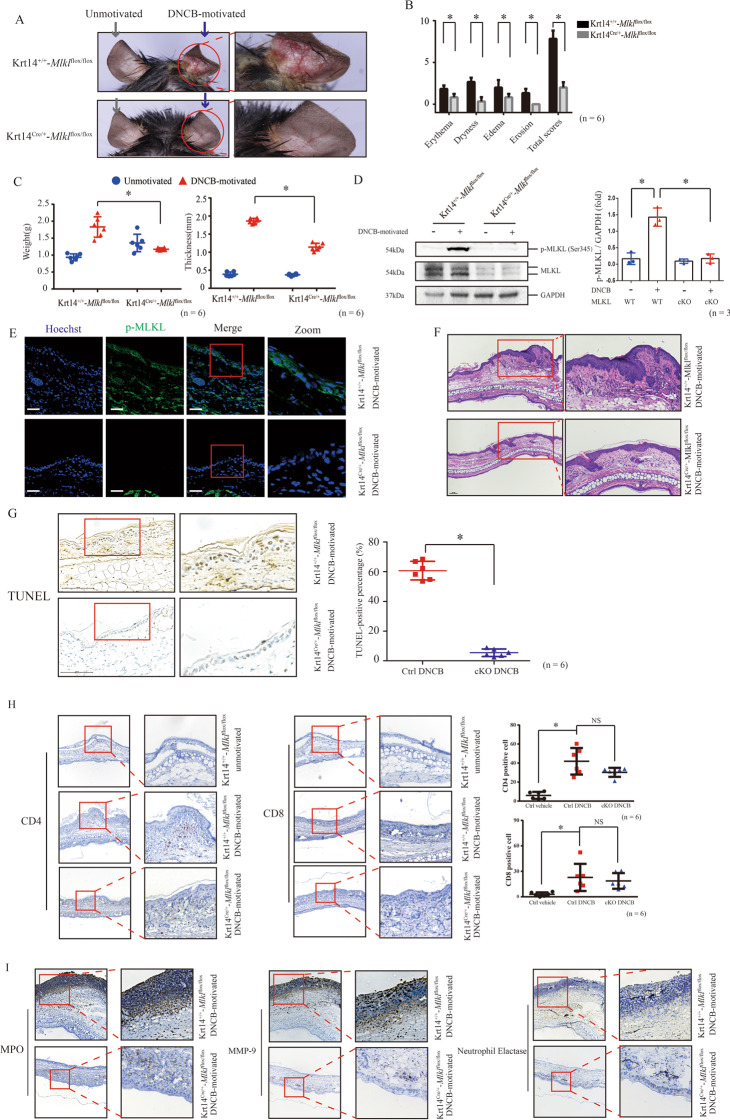
MLKL-mediated keratinocyte necroptosis is required in DNCB-induced CHS. **A** The skin appearance of *Mlkl* cKO mice (Krt14^Cre/+^-*Mlkl*^flox/flox^) and the control mice (Krt14^+/+^-*Mlkl*^flox/flox^) after DNCB motivation. Left ear was the sample of unmotivated tissue (gray arrow), and right ear was motivated by DNCB (blue arrow). **B**, **C** The skin manifestation of the cKO and control mice was evaluated by Dermatitis Score, ear weight and thickness. (*n* = 6). **D** The protein level of MLKL in the epidermis of cKO mice was significantly decreased. Statistical analysis of the interested protein was shown (*n* = 3). **E** Phosphorylation of MLKL cannot be observed in the DNCB-motivated ear of cKO mice in the assay of immunofluorescence staining (green). (*n* = 6). Scale bar represents 100 μm. **F** The histology feature of DNCB-motivated ear was showed by the tissue section staining with H&E staining (20× magnification). **G** The level of keratinocyte death in skin necrosis was determined by TUNEL staining. (*n* = 6). Scale bar represents 100 μm. **H** Infiltration of CD4 + , CD8 + cells in ear tissue of different group in *Mlkl* cKO mice and control mice were detected by immunohistochemistry assay. Quantitative analysis of CD4 + and CD8 + cells in the skin tissues was showed. (*n* = 6). **I** The neutrophils activation indicators, MPO, MMP-9 and NE in the DNCB-motivated ears of *Mlkl* cKO mice and control mice were detected by immunohistochemistry assay. ∗*p* < 0.05.

We established keratinocyte-specific *Mlkl* cKO mice to explore the role of MLKL-mediated keratinocyte necroptosis in pathogenesis of DNCB-motivated CHS. We observed that the control mice (Krt14^+/+^-*Mlkl*^flox/flox^) presented similar dermatitis lesions as wild type mice, but we did not observe dermatitis skin appearances in *Mlkl* cKO mice (Krt14^Cre/+^-*Mlkl*^flox/flox^) (Fig. 2A-C). Moreover, we validated that MLKL phosphorylation was increased in control mice but not in cKO mice after DNCB motivation (Fig. 2D and E). Furthermore, we had not observed similar histological features of dermatitis in epidermis of DNCB-motivated cKO mice as control mice (Fig. 2F). Importantly, counts of TUNEL-positive keratinocytes of DNCB-motivated cKO mice were significantly lower than control (Fig. 2G).

In CHS, hapten is presented to T cells by Langerhans cells and dendritic cells. When re-encountered, activated hapten-specific T cells will be chemotactic and recruited to initial exposure site, releasing cytokines that stimulate keratinocytes, which then induce a chain reaction of inflammation [18]. We found that although the infiltration of CD4 + and CD8 + lymphocytes still can be observed in DNCB-motivated ear of cKO mice (Fig. 2H). There is no difference in infiltration levels of CD4 + and CD8 + lymphocytes between cKO mice and control mice after DNCB motivation, suggesting that lymphocytes were still recruited into CHS skin lesion. Importantly, we observed that the high levels of neutrophils activation indicators [19, 20] myeloperoxidase (MPO), matrix metalloprotein-9 (MMP-9), and Neutrophil Elactase (NE) were significantly inhibited in the DNCB-motivated ears of cKO mice, compared with that of control mice (Fig. 2 I).

Therefore, these findings indicated that epidermal keratinocyte-specific MLKL deficiency suppresses DNCB-motivated skin lesion in CHS mice.

### Inhibiting necroptosis by specific inhibitors retrieves DNCB-motivated CHS

**Fig. 3:**
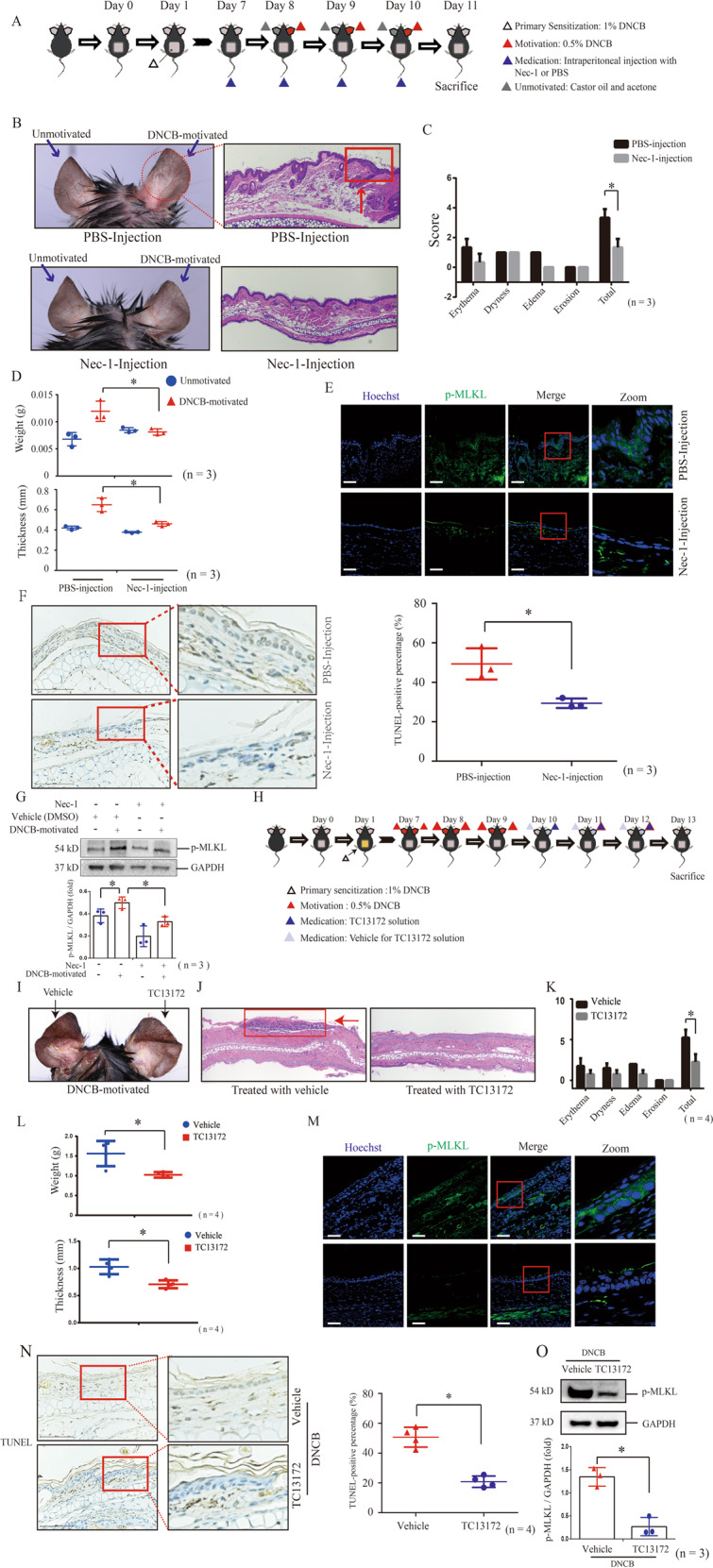
The treatment of inhibitors targeting MLKL-mediated necroptosis relieves DNCB-motivated dermatitis. **A** Flowchart of the treatment of necrostatin-1 for CHS murine model. **B** CHS mice were received intraperitoneal injection of 100 μl Nec-1 (10 μM) or solvent PBS at the day before motivated by DNCB. The histology feature was showed by H&E staining (20× magnification). **C**, **D** The severity of skin manifestation was evaluated by Dermatitis Score, ear weight and thickness. (*n* = 3). **E** The phosphorylation of MLKL was detected by immunofluorescence staining (green). Scale bar represents 100 μm. **F** The level of keratinocyte death was determined by PI staining. (*n* = 3). **G** The protein level and phosphorylation of MLKL were detected by western blotting. Statistical analysis of the interested protein was shown (*n* = 3). **H** Flowchart of the treatment of TC13172 for CHS murine model. **I** The skin appearance of CHS mice before and after the topical application of TC13172 and vehicle. **J** The histology features were showed by H&E staining (20× magnification). **K**, **L** The severity of skin manifestation was evaluated by Dermatitis Score, ear weight and thickness. (*n* = 3). **M** The phosphorylation of MLKL was detected by immunofluorescence staining (green) (*n* = 3). Scale bar represents 100 μm. **N** The level of keratinocyte death was determined by TUNEL staining. (*n* = 3). **O** The protein level and phosphorylation of MLKL were detected by western blotting. Statistical analysis of the interested protein was shown (*n* = 3). ∗*p* < 0.05.

We found that intraperitoneal injection of necrostatin-1 (a general-used necroptosis inhibitor targeting RIPK1 [21]) retrieved skin dermatitis of DNCB-motivated mice (Fig. 3A-D). Furthermore, we observed that MLKL phosphorylation and TUNEL positive cells in epidermis of DNCB-motivated ears of necrostatin-1-injected mice were lower than those of solvent PBS-injected mice (Fig. 3E and F). We also validated that increase of MLKL phosphorylation in DNCB-motivated ears can be partly inhibited by necrostatin-1 injection (Fig. 3G).

TC13172 is one necroptosis inhibitor targeting MLKL [22]. We found that topical application of TC13172 retrieved skin manifestation and histological changes of DNCB-motivated dermatitis (Fig. 3H-L). Its treatment also inhibited the increases of MLKL phosphorylation and TUNEL staining after DNCB motivation (Fig. 3M-O).

These findings demonstrate that treatment of necroptosis inhibitors can retrieve DNCB-motivated dermatitis, further indicating that MLKL-mediated necroptosis contributes the development of DNCB-motivated CHS.

### Topical application of dexamethasone inhibits the keratinocyte death and activation of necroptosis regulators in CHS lesion

**Fig. 4:**
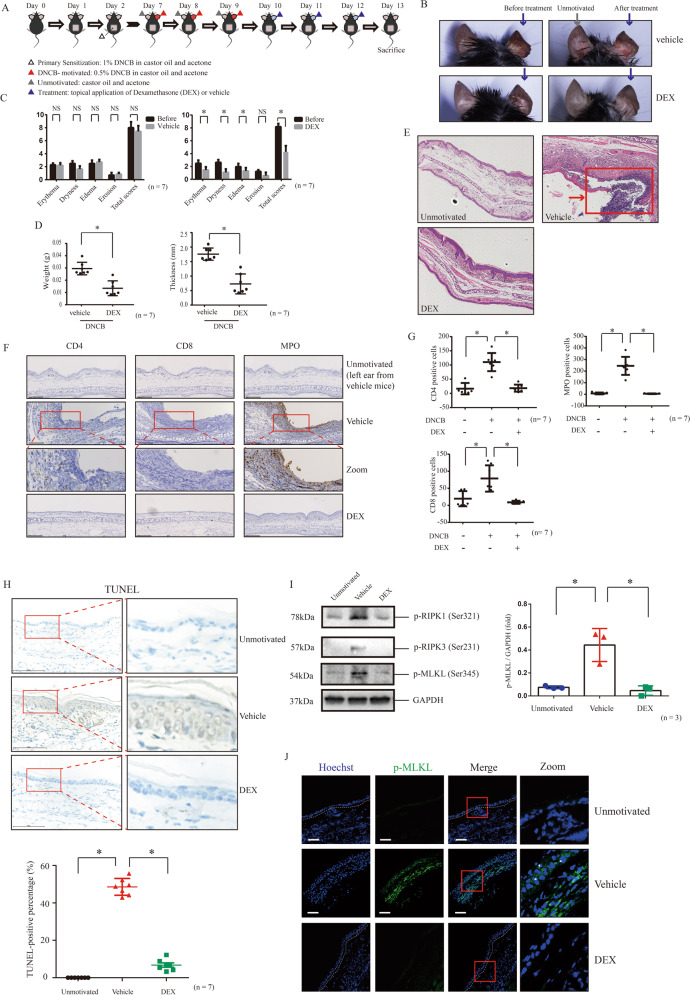
The treatment of dexamethasone reduced keratinocytes necroptosis and relieved DNCB-motivated dermatitis. **A** Flowchart of the treatment of dexamethasone for CHS murine model. **B** The skin appearance of CHS mice before and after the topical application of vehicle or dexamethasone. The samples of unmotivated tissue were taken from the left ears of the mice in the vehicle group (indicated by gray arrow). Other tissue samples were indicated by blue arrow. **C**, **D** The improvement of skin manifestation after the treatment of dexamethasone was evaluated by Dermatitis Score, ear weight and thickness. (*n* = 7). **E** The histology feature of tissue from different group of mice was showed by H&E staining (20× magnification). Red arrow indicated skin necrosis. ∗*p* < 0.05. **F** CD4 + , CD8 + or MPO + cells infiltrating in ear tissue of different mice group were detected by immunohistochemistry assay. Scale bar represents 150 μm. **G** Quantitative analysis of CD4 + , CD8 + or MPO + cells in the skin tissues from the different mice group. (*n* = 7). ∗*p* < 0.05. **H** The level of keratinocyte death was determined by TUNEL staining. (*n* = 7). **I** The phosphorylation levels of RIPK1, RIPK3 and MLKL in mice treated with topical dexamethasone therapy were detected by western blotting. Statistical analysis of the interested protein was shown (*n* = 3). **J** The phosphorylation of MLKL was detected by immunofluorescence staining (green). (*n* = 7). Scale bar represents 100 μm. ∗*p* < 0.05.

Topical corticosteroid therapy is generally used in treatment for ACD and atopic dermatitis [23, 24]. Thus, we explored whether keratinocyte death and activation of necroptosis regulators can be retrieved after topical application of dexamethasone. We validated that topical dexamethasone therapy decreased total scores of dermatitis, and weight and thickness of DNCB-motivated ears (Fig. 4A-D). Histologically, treatment of dexamethasone retrieved epidermal necrosis and inflammatory cell infiltration (CD4 + lymphocytes, CD8 + lymphocytes and MPO + cells) in DNCB-motivated ears (Fig. 4E-G). Furthermore, we found that dexamethasone decreased TUNEL positive cells count (Fig. 4H). We observed that treatment of dexamethasone inhibited the increase of MLKL, RIPK3 and RIPK1 phosphorylation in the lysis of DNCB-motivated ears (Fig. 4I). We also validated that dexamethasone treatments inhibited the increase of MLKL phosphorylation in DNCB-motivated ears through immnunofluorescence staining (Fig. 4J).

These findings suggest that topical application of dexamethasone could retrieve keratinocyte death and activation of necroptosis regulators in DNCB-induced CHS lesion.

### Treatment of dexamethasone inhibits MLKL-mediated necroptosis in cultured keratinocytes

**Fig. 5:**
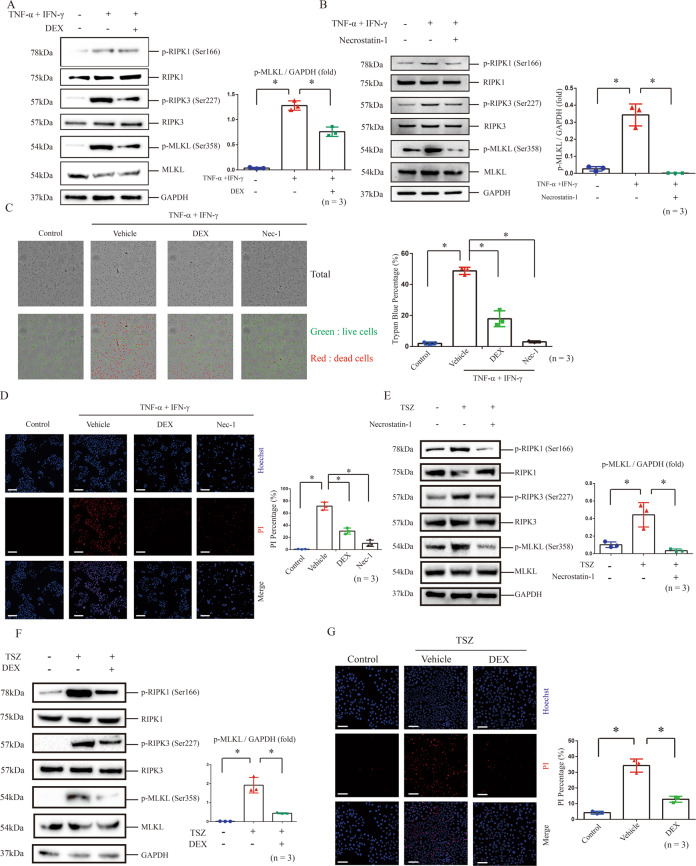
Dexamethasone or necroptosis inhibitor necrostatin-1 inhibits necroptosis in TNF-α and IFN-γ- treated HaCaT cells. **A**, **B** The protein level and phosphorylation of RIPK1, RIPK3 and MLKL were detected by western blotting in the TNF-α and IFN-γ- treated HaCaT cells in the presence or absence of dexamethasone (**A**), as well as necrostatin-1 (**B**). Statistical analysis of the interested protein was shown (*n* = 3). Trypan blue staining (**C**) and PI staining (**D**) were used to determine the levels of cell death in TNF-α and IFN-γ- treated HaCaT cells in the presence or absence of dexamethasone or necrostatin-1. The photos of HaCaT cells in (**C**) are 20× magnification. And the threshold is 4–30 μm. Scale bar (**D**) represents 100 μm. ∗: *p* < 0.05. **E**, **F** Necroptosis was induced by the treatment of TNF-α (100 ng/ml), Smac mimetic compound (100 nM) and z-VAD-fmk (20 μM) (TSZ) in HaCaT cells for 12 h, and protein level and phosphorylation of RIPK1, RIPK3 and MLKL were detected by western blotting. Necrostatin-1 treatment was used to validate the inhibition of necroptosis. Statistical analysis of the interested protein was shown (*n* = 3). **G** PI staining was used to determine the levels of cell death in TSZ- treated HaCaT cells in the presence or absence of dexamethasone. Scale bar represents 100 μm.

As described previously [25, 26], HaCaT cells treated with TNF-α and IFN-γ were used to simulate the stimulation by which keratinocytes were suffered in immune microenvironment of CHS (Fig. 5A-D). We found that phosphorylation of MLKL, RIPK3 and RIPK1 was increased in HaCaT cells treated by TNF-α and IFN-γ (Fig. 5A). Increased phosphorylation of these necroptosis core regulators can be inhibited by the treatment of dexamethasone or necrostatin-1 (Fig. 5A-B). Next, we found both treatments inhibited the increase of trypan blue or PI- positive cells in HaCaT cells challenged by TNF-α and IFN-γ (Fig. 5C and D).

We evaluated the response of HaCaT cells to necroptosis induction by combination treatment with TNF-α, Smac mimetic compound and z-VAD-fmk (TSZ), which is an effective method to induce necroptosis [27]. We validated that phosphorylation levels of MLKL, RIPK3 and RIPK1 were increased after TSZ treatment (Fig. 5E). Phosphorylation of MLKL, RIPK3 and RIPK1 as well as increase of PI-positive cells induced by TSZ treatment can be inhibited by dexamethasone (Fig. 5F-G).

These findings indicated that inhibiting keratinocyte necroptosis might be involved in therapeutic effects of dexamethasone against the DNCB-induced CHS.

### MLKL-mediated HMGB1 secretion from keratinocyte facilitates DNCB-motivated CHS

**Fig. 6:**
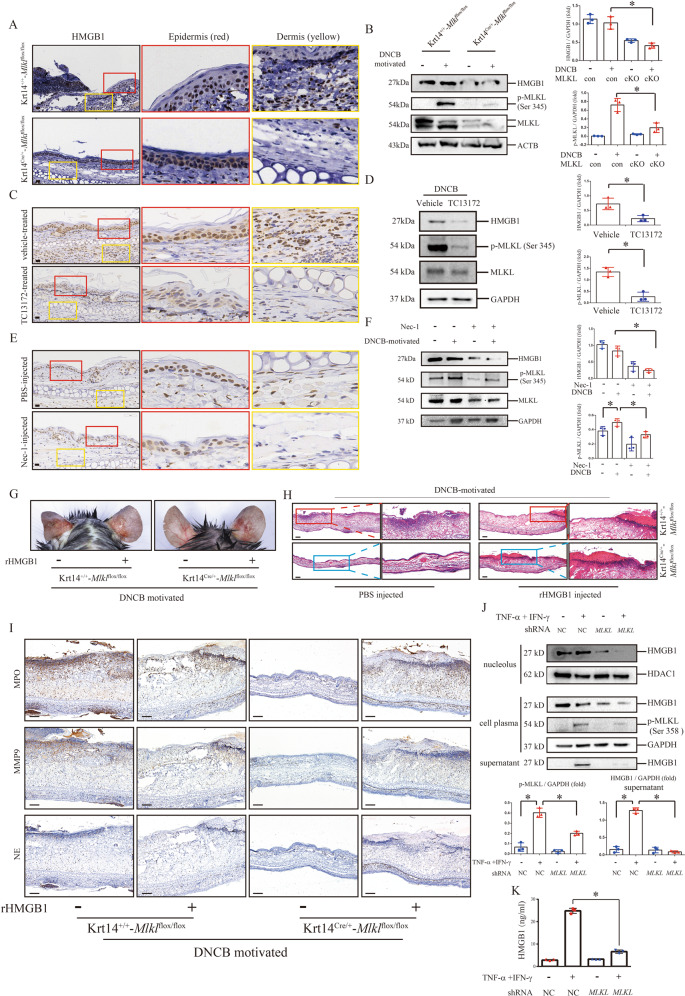
MLKL-mediated HMGB1 secretion from keratinocyte facilitates DNCB-motivated CHS. **A** HMGB1 in the DNCB-motivated ears of *Mlkl* cKO mice and control mice were detected by immunohistochemistry assay. **B** The protein level of HMGB1 in epidermis was detected by western blotting. Statistical analysis of the interested protein was shown (*n* = 3). **C** HMGB1 in the DNCB-motivated ears of wild type mice treated by intraperitoneal injection with TC13172 or solvent PBS were detected by immunohistochemistry assay. **D** The protein level of HMGB1 in epidermis was detected by western blotting. Statistical analysis of the interested protein was shown (*n* = 3). **E** HMGB1 in DNCB-motivated ears of wild type mice topically treated by necrostatin-1 or vehicle were detected by immunohistochemistry assay. **F** The protein level of HMGB1 in epidermis was detected by western blotting. Statistical analysis of the interested protein was shown (*n* = 3). **G**–**I** *Mlkl* cKO mice (Krt14^Cre/+^-*Mlkl*^flox/flox^) and control mice (Krt14^+/+^-*Mlkl*^flox/flox^) were intradermal injected with rHMGB1 (right ear) and PBS (left ear), respectively. The skin appearance (**G**), histology feature (**H**) and infiltration of MPO, MMP-9 and NE (**I**) were shown. **J** The protein levels of HMGB1 in nucleus, cell plasma and supernatant were detected by western blotting in the MLKL knockdown HaCaT cells in the presence or absence of TNF-α and IFN-γ. Statistical analysis of the interested protein was shown (*n* = 3). **K** HMGB1 secretion in supernatants of cultured HaCaT cells were determined by ELISA. Scale bar (**A**, **C**, **E**) represents 20 μm. Scale bar (**H**, **I**) represents 200 μm. ∗*p* < 0.05.

As DAMPs, HMGB1 released from keratinocytes was discovered to contribute psoriatic skin inflammation through the crosstalk between HMGB1 autosecretion and T cells [11]. We found that infiltrated inflammatory cells in DNCB-motivated ears of Krt14^+/+^-*Mlkl*^flox/flox^ mice expressed high level of HMGB1, which cannot be observed in *Mlkl* cKO mice (Fig. 6A). HMGB1 was decreased in lysate of DNCB-motivated ears of cKO mice, compared with those of control mice (Fig. 6B). We found that DNCB-induced abnormal expression of HMGB1 in infiltrated inflammatory cells was inhibited by either topical application of TC13172 or necrostatin-1 injection (Fig. 6C and E), and its protein level in tissue lysate of DNCB-motivated ear was also decreased after two treatments (Fig. 6D and F).

Intradermal injection of rHMGB1 led to reappearance of CHS inflammation in DNCB-motivated *Mlkl* cKO mice (Fig. 6G, H, S1A, B). The increase of MPO, MMP-9 and NE, and PI positive cells count in epidermal was also reappeared in these mice ears (Fig. 6I, S1C).

To explore the role of MLKL in HMGB1 releasing from keratinocytes, we established MLKL knockdown HaCaT cells to detect the production and secretion of HMGB1 after stimulation of TNF-α and IFN-γ (Fig.S1E). We observed that HMGB1 was elevated in supernatant of HaCaT cells after stimulation (Fig. 6J). Meanwhile, its level was decreased in cell plasma, indicating increase of secretion from intracellular to extracellular (Fig. 6J). HMGB1 was decreased in nucleus, cell plasma, and supernatant of TNF-α and IFN-γ-challenged knockdown HaCaT cells, compared with control (Fig. 6J). Through ELISA assay, we validated that increase of HMGB1 secretion after stimulation was inhibited in knockdown HaCaT cells (Fig. 6K).

These findings indicate that MLKL-mediated keratinocytes necroptosis promotes and amplified skin inflammation by increasing HMGB1 secretion in CHS.

## Discussion

Our study demonstrates that MLKL-mediated necroptosis contributes epidermal damage in microenvironment of DNCB-induced CHS, and might develop into a potential therapeutic target.

The identification of RIPK3 as necroptosis core regulator is the key step in demonstrating mechanism how cells run necroptosis process [27–29]. Furthermore, MLKL was identified as one RIPK3 substrate, and its activation is required for executing RIPK3-mediated necroptosis [21, 30]. MLKL directly or indirectly leads to cell membrane damage through its N-terminal four-helical bundle domain [31–33]. These findings greatly contributed to unraveling the regulatory mechanism of necroptosis. Here, we observed MLKL activation in keratinocytic necrosis of CHS. Importantly, keratinocytic necrosis and manifestations were inhibited in keratinocyte-specific *Mlkl* cKO mice. These findings indicate that MLKL-mediated keratinocyte necroptosis is required for pathogenesis of CHS.

Bonnet et al. [34] reported that keratinocyte-specific FADD cKO mice (FADD^E-KO^ mice) present severe phenotype of skin inflammation. They found that FADD^E-KO^*Ripk3*^-/-^ mice did not exhibit skin inflammation development. Dannappel et al. [35] found that apoptosis and necroptosis were triggered in keratinocytes of RIPK1^E-KO^ mice. These mice presented the severe skin inflammation. Deficiency of either RIPK3 or MLKL prevented development of skin inflammatory lesion in RIPK1^E-KO^ mice. These findings validate that keratinocyte necroptosis regulation plays a crucial role on constraint of skin inflammation and maintenance of skin homeostasis. Our study supports this paradigm, as in addition to preventing RIPK1 deficiency-caused spontaneous skin inflammation, keratinocyte-specific MLKL deficiency also prevented exogenous disturbance-induced skin inflammation.

Necroptosis was reported to involve in some kinds of skin disorders [36] such as infection of Staphylococcus aureus [37] or Herpes simplex virus [38], Toxic Epidermal Necrolysis (TEN) [39], systemic lupus erythematosus [40], lichen planus [41], and psoriasis [42]. Significant keratinocyte death and separation on the junction between epidermis and dermis are typical histological features of TEN [43]. Viard-Leveugle et al. [44] reported that drug-activated T cells of TEN patient secreted high level of TNF-α and IFN-γ. Kim et al. [39] reported that RIPK3 protein and MLKL phosphorylation were increased in skin lesion of TEN. Their data suggest that TNF-α and IFN-γ are key triggers in initiation of keratinocyte necroptosis.

In microenvironment of different diseases, HELPER T-cells can finely regulate the balance between Th1 and Th2 immune responses. Their imbalance results in pathogenesis of many kinds of diseases. TNF-α and IFN-γ are Th1-assocaited cytokines, [45] and Lauffer et al. [41] reported that lichen planus and lupus erythematosus, both of which present histopathological feature of interface dermatitis, shared the significant transcriptomics characteristics of type I immune response. They found that T cells infiltrating in skin lesions were dominated by TNF-α and IFN-γ positive cells. Mixed supernatant of T cells derived from the lesions of lichen planus and lupus erythematosus induced phosphorylation of RIPK3 and MLKL in keratinocytes, but the inducing effect is dependent on the presence of TNF-α and IFN-γ in supernatant. Th1 cells contribute to the pathogenesis of CHS, and play a pro-inflammatory effect through producing IFN-γ and other pro-inflammatory cytokines [46–49]. Our study verified that Th1 immune microenvironment of CHS initiates necroptosis in keratinocytes, which plays a crucial role in promoting epidermal damage.

We had observed the similar level of infiltration of T cells (CD4 + and CD8 + ) in DNCB-motivated ears of *Mlkl* cKO mice as control mice, but they had not presented skin appearance of CHS. We speculate that suppression of keratinocyte necroptosis in response to the stimulation from T cells infiltrating after DNCB-motivation is the key reason for epidermal damage silence. We noticed that infiltration and activation of neutrophils were decreased in DNCB-motivated *Mlkl* cKO mice, compared with DNCB-motivated control mice. We speculate that through running necroptosis, keratinocyte might function as a self-amplification effector between infiltrating T cells-derived stimulation and epidermal damage caused by activation of neutrophils in CHS immune microenvironment. Activation of neutrophils by keratinocytes undergoing necroptosis leads to aggravation of epidermal damage and further recruitment of inflammatory cells. Therefore, we can observe a significant reduction of neutrophils infiltration in *Mlkl* cKO mice after DNCB motivation, although T cells infiltration can still be found.

During process of necroptosis, DAMPs would be released from necrotic cells, [50] and participate regulation of immune responses. We speculate that abolishment of MLKL-mediated keratinocyte necroptosis blocks DAMPs secretion and their contribution in self-amplification of immune responses in epidermis. As one DAMPs, HMGB1 was observed to be decreased by treatments of inhibiting MLKL-mediated keratinocyte necroptosis in this study. Wang et al’s [11] found that HMGB1 exhibited the most effect among autosecretory proteins involving psoriasiform dermatitis. Therefore, we speculate that HMGB1 might be the key DAMPs from keratinocytes in the development of CHS.

In summary, we report that MLKL-mediated keratinocyte necroptosis plays a crucial role in epidermal damage in inflammatory microenvironment of CHS. Furthermore, MLKL-mediated necroptosis deficiency or inhibition resulted in blocking recruitment and activation of inflammatory cells in CHS via reducing HMGB1 release in keratinocytes. However, we did not clearly clarify the mechanism that regulates keratinocyte necroptosis in immune microenvironment of CHS. Our study provides the novel evidence that MLKL-mediated keratinocyte necroptosis is a promising pharmaceutic target for dermatitis of CHS.

## Materials and Methods

### Reagents

Dexamethasone (#D4902) and 2,4-dinitrochlorobenzene (DNCB, #138630) were purchased from Sigma-Aldrich (St. Louis, MO, USA). Castor oil and acetone were purchased from Aladdin Chemical (Shanghai, China) and Sinopharm Chemical Reagent (Shanghai, China), respectively. Propidium iodide (PI) / RNase staining solution (#4087 S) was purchased from Cell Signaling Technology (Danvers, MA, USA). In this study, some antibodies were purchased from Cell Signaling Technology, including phospho-RIP (#65746 for human and #38662 for mouse), RIP3 (#95702), Phospho-RIP3(#91702), MLKL (#37705), phospho-MLKL (#37333), GAPDH (#5174), and goat anti-rabbit IgG HRP-linked antibody (#7074). Other antibodies were purchased from Abcam (Cambridge, MA, USA), including RIP (#ab125072, #ab72139), RIP3 (#ab226297), Phospho-RIP3 (#ab209384), MLKL (#ab184718), phospho-MLKL (#ab187091), CD4 (#ab183685), CD8 (#ab217344), MPO (#ab208670), MMP-9 (#ab283575) and NE (#ab68672). Tumor necrosis factor α (TNF-α), Smac-mimetic compound, z-VAD-fmk and necrostatin-1 (as described previously [27]) were gifts from Dr. Sudan He’s Lab in Suzhou Institute of Systems Medicine. TNF-α and IFN-γ used to simulate the ACD microenvironment in HaCaT were purchased from R&D system (Minneapolis, USA) and PeproTech (New Jersey, USA). rHMGB1 was purchased from R&D system (Minneapolis, USA).

### Animals

All mice were purchased from GemPharmatech (Nanjing, Jiangsu, China). A total of 57 wild type C57BL/6 J mice aged 6 to 8 weeks (15-25 g) were included. Mice were allocated to the groups randomly. Keratinocyte-specific *Mlkl* conditional knockout (cKO) mice (Krt14^Cre/+^-*Mlkl*^flox/flox^,) were the offspring mice intercrossing from B6/JGpt-*Mlkl*^*em1Cflox*^/Gpt mice and B6/JGpt-H11^em1Cin(hKRT14-iCre)^/Gpt mice. The control mice (Krt14^+/+^-*Mlkl*^flox/flox^) were also the offspring of B6/JGpt-*Mlkl*^*em1Cflox*^/Gpt mice and B6/JGpt-H11^em1Cin(hKRT14-iCre)^/Gpt mice, and served as the control of the *Mlkl* cKO mice.

We performed experiments of mice in specific pathogen-free facilities of Nanjing Medical University. Our animal experiment was approved by Institutional Animal Care and Use Committee of Nanjing Medical University (Approval No. IACUC-2101043). The procedures of establishing animal model are described in flowcharts of Figs. 1C, 3A, 3H and 4A.

### Preparation of Medications in animal studies

DNCB was dissolved in 0.5% and 1% (w/v) solution using a 1:4 volume ratio of DNCB *versus* mixture of acetone and castor oil.

Formulation of TC13172 solution (0.25%) is described in Table 1. O/W cream base formulation of dexamethasone (0.75%) is described in Table 2.

**Table 1:** The formulation of TC13172 solution (0.25%).

Ingredients	Content (%)
API	0.25
Glycerin	25
Sodium carboxymethylcellulose	1
Distilled water	25
DMSO	48.75

**Table 2:** The O/W cream base formulation of dexamethasone (0.75%).

Phase of cream base formulation	Ingredients	Content (%)
Oil phase	Liquid paraffin	3
	White Vaseline	5
	Octadecanol	1.5
	Glyceryl mono-stearate	3
Water phase	Propanediol	0.5
	Distilled water	33.4
	PEG400	2.5
	Ethylparaben	0.1
	Cocamidopropyl Betaine	1

### Dermatitis Scores

The details are described in the Table 3. Two investigators blinded to the specific group evaluated dermatitis scores and the mean of their scores was calculated.

**Table 3:** Measurement of Dermatitis Score.

Score	Erythema	Scale	Edema	Erosion
0 (None)	Very similar to unmotivated			
1 (Mild)	Angiotelectasis	Dryness	Swelling of the ear root	Mild erosion
2 (Moderate)	Erythema	Dot-like Scale	Half of the ear swollen	Exudation
3 (Severe)	Hemorrhage	LamellarScale	Entire ear swollen	Exfoliation or Crust

### Cell Culture

Human keratinocytes cell line HaCaT cells (China Center for Type Culture Collection) were cultured in Dulbecco’s modified Eagle’s medium containing 10% fetal bovine serum (Gibco, CA, USA). Cells have been authenticated by STR profiling.

### Immunofluorescence staining

Immunofluorescence staining was performed as described previously [51].

### Western blotting assay

Western blotting assay was performed as previously described [51]. The the density of interested protein bands was quantified by ImageJ software.

### Immunohistochemistry study

Immunohistochemistry study was performed as described previously [51].

### Counting of CD4 + , CD8 + , MPO + or TUNEL-positive cells

We randomly selected six different regions for each tissue section at 40× magnification under light microscope. Then, we counted CD4 + , CD8 + , MPO + or TUNEL-positive cells in each region by ImageJ. The average count of positive cells for six regions from each tissue section represented infiltration levels of CD4 + , CD8 + or MPO + cells.

### PI and TUNEL staining

PI staining was done as previously described [51]. Briefly, cells were cultured in dishes with glass bottom. Cells were fixed with paraformaldehyde and stained with PI. TUNEL staining was performed with a TUNEL kit (Shanghai Recordbio Biological Technology, Shanghai, China) according to the manufacturer’s instructions.

### Counting of trypan blue positive cells

After indicated treatment, HaCaT cells were digested and resuspended. Then, trypan blue (Gibco) was added to the cell solution according to manufacturer’s instructions. The number of viable and dead cells were differentiated by trypan blue exclusion and counted by Corning Cell Counter (Corning, NY, USA) using CytoSMART software (CytoSMART Technologies, Eindhoven, Netherlands). Threshold: 4–30 μm.

### Mlkl knockdown cell line establishment

Lentiviruses carrying NC or MLKL shRNAs were obtained from GenePharma (Shanghai, China). NC shRNA sequence: 5′-TTCTCCGAACGTGTCACGT-3′ MLKL shRNA sequence: 5′-GGAGCTCTCGCTGTTACTTCA-3′ (742); 5′-GGAGATCCCGCAAGAGCAAAT-3′ (970); 5′-GCAATAGTGAGGCAGACTTTC-3′ (1133). To obtain stable MLKL knockdown HaCaT cells, the cells were selected by puromycin incubation.

### Protein extraction from nucleus and cytoplasm

NE-PER™ nuclear and cytoplasmic extraction reagents (Thermo Fisher Scientific, Waltham, MA, USA) was used to extract protein sample from nucleus and cytoplasm separately as previous [51, 52].

### Protein extraction from supernatant of cultured cells

We added methanol and chloroform into supernatant of cultured HaCaT cells to extract protein. After shaking and centrifugation, top layer of liquid was discarded. Precipitate was lyophilized to powder under vacuum.

### ELISA assay

According to manufacturer’s instructions, HMGB1 in supernatant of cultured HaCaT cells was determined by ELISA kit (Chondrex, WA, USA).

### Statistical analysis

Student’s *t*-test or adjusted *t*-test were used to analyze the differences between two groups. One way ANOVA followed by Tukey’s test or Kruskal–Wallis test followed by Dunn’s post hoc test was used to analyze the differences among three or more groups. Data was obtained from at least three of independent experiments. No statistical method was used to predetermine the sample size. The variance is similar between the groups for statistically comparison. All data were presented as mean ± SD. Statistical significance was identified as *p* < 0.05.

### Injection of recombinant HMGB1

*Mlkl* cKO mice and the control mice were both sensitized and motivated with DNCB. One day before DNCB motivation (Day 6), rHMGB1 (1 μg) or PBS was intradermal injected in ears. We validated that simply intradermal injection of rHMGB1 in mice ears did not induce skin inflammation (Fig S1D).

## Supplementary information

Supplementary information

Original Data File

## Data Availability

The datasets analyzed in this study are available through the accession number GSE6281 from the Gene Expression Omnibus (GEO) database (http://www.ncbi.nlm.nih.gov/geo/). All data in this manuscript could be requested from corresponding authors.
