# Supplementary material for: Necroptosis-mediated HMGB1 secretion of keratinocytes as a key step for inflammation development in contact hypersensitivity
Source: Cell Death Discov. 2022 Nov 7;8:451. doi: 10.1038/s41420-022-01228-6 (PMC9640721; doi:10.1038/s41420-022-01228-6)
Supplement: Supplementary file 1 — Supplementary information [file 41420_2022_1228_MOESM1_ESM.docx]

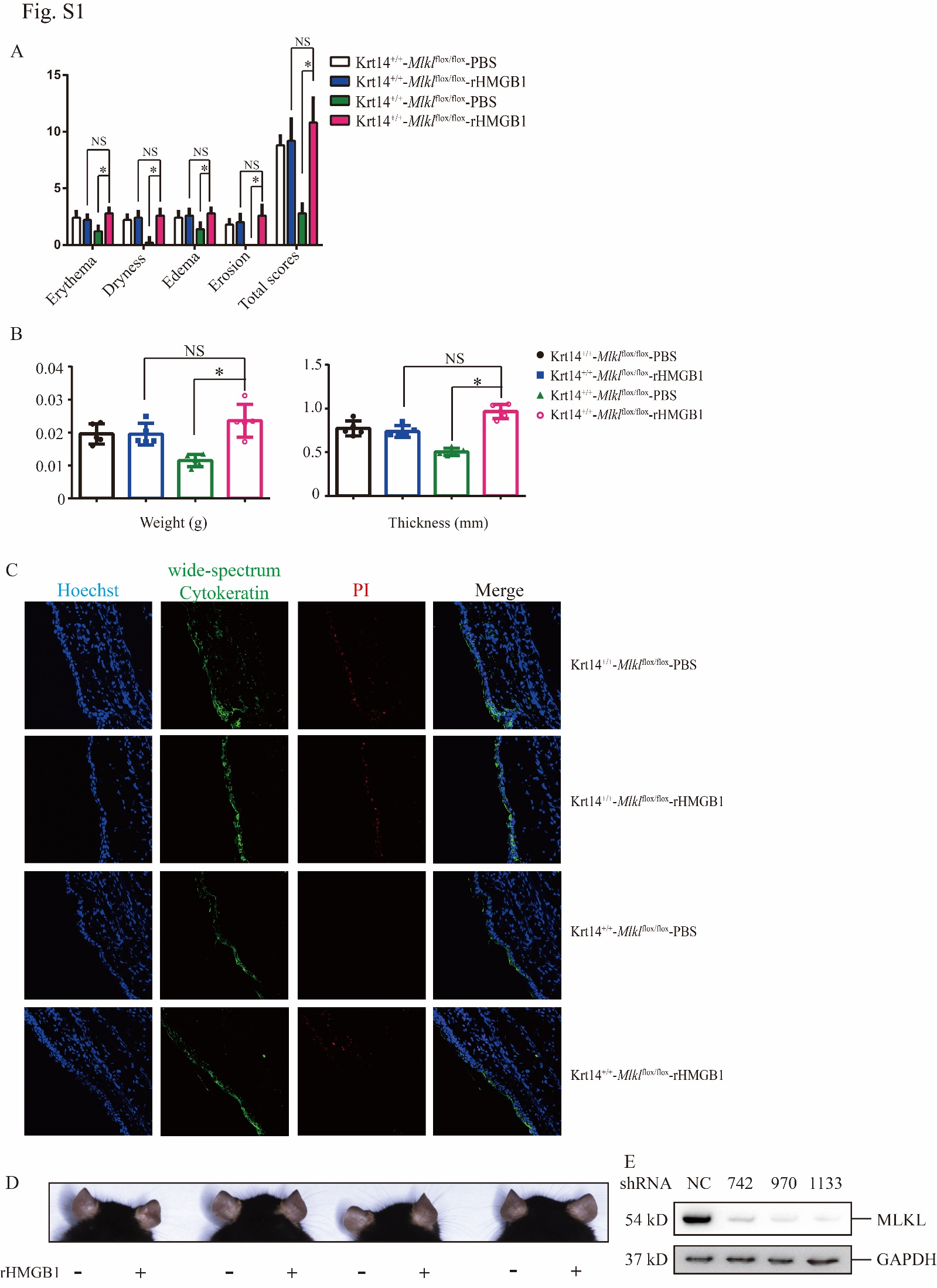


**Figure S1**

(A): The skin appearance compared with the ACD mice received intracutaneous injection of recombinant HMGB1and vehicle (PBS). (B): The skin manifestation of different mice group was evaluated by Dermatitis Scoring and the assaying for the weight and thickness of ears. ∗ : *p* < 0.05. n = 3. (C): The level of keratinocyte death was determined by PI staining. n = 3. (D): Establishment of MLKL knockdown HaCaT cells by transfecting with lentiviruses carrying MLKL shRNA. The efficiency of knockdown was examined by Western blotting.
