# Supplementary material for: Necroptosis-mediated HMGB1 secretion of keratinocytes as a key step for inflammation development in contact hypersensitivity
Source: Cell Death Discov. 2022 Nov 7;8:451. doi: 10.1038/s41420-022-01228-6 (PMC9640721; doi:10.1038/s41420-022-01228-6)

Source data of Figure 1H

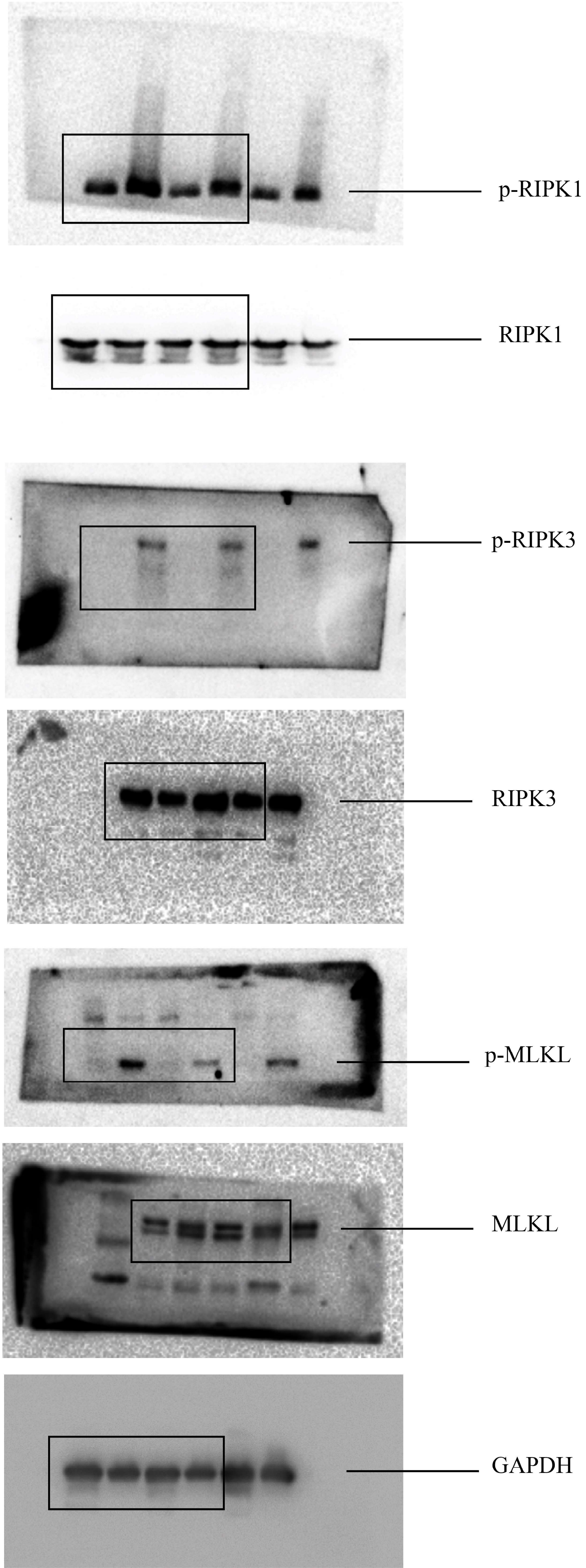

Source data in Figure 2D

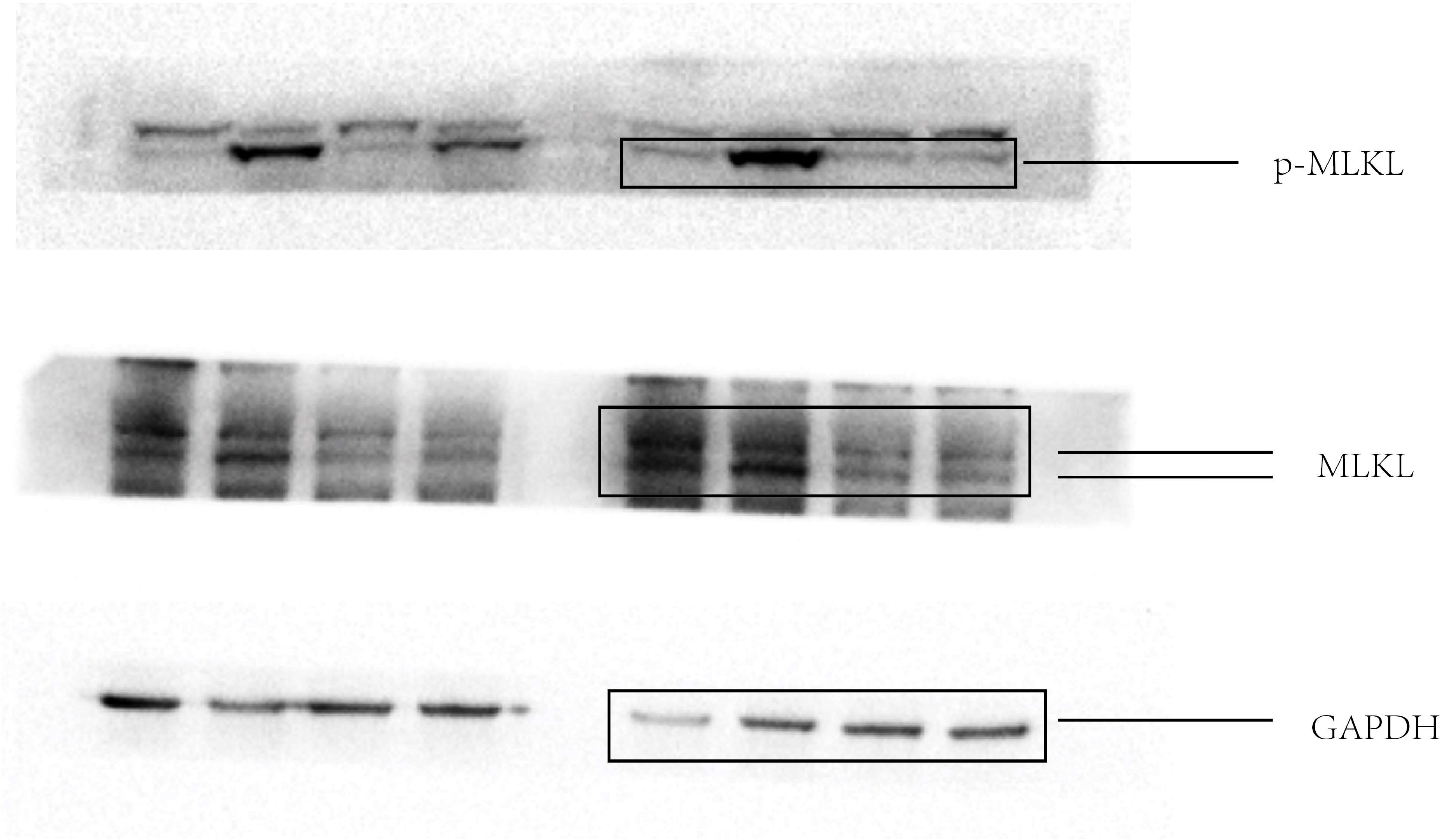

Source data in Figsure 3G

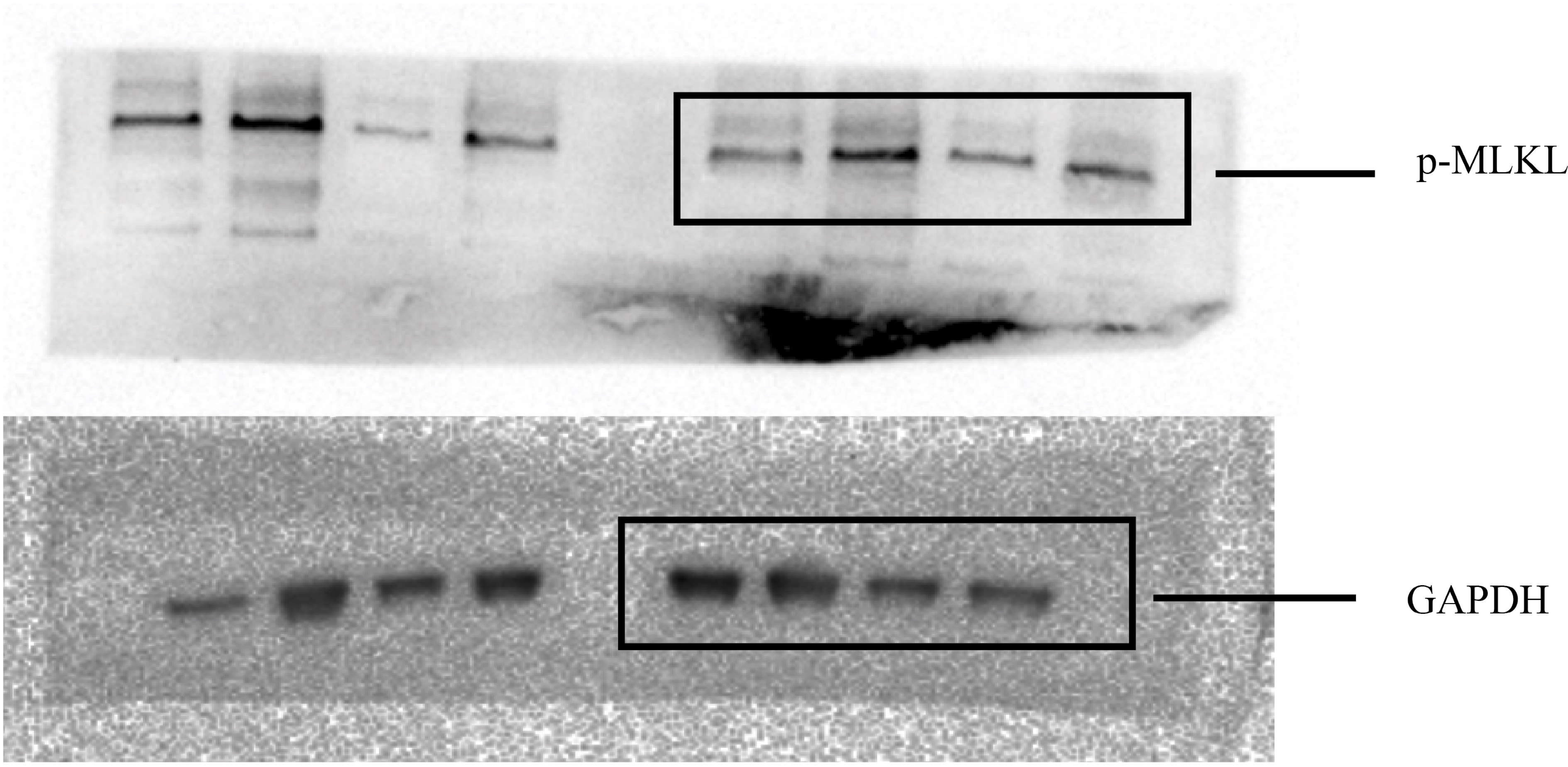


Source data in Figsure 3O

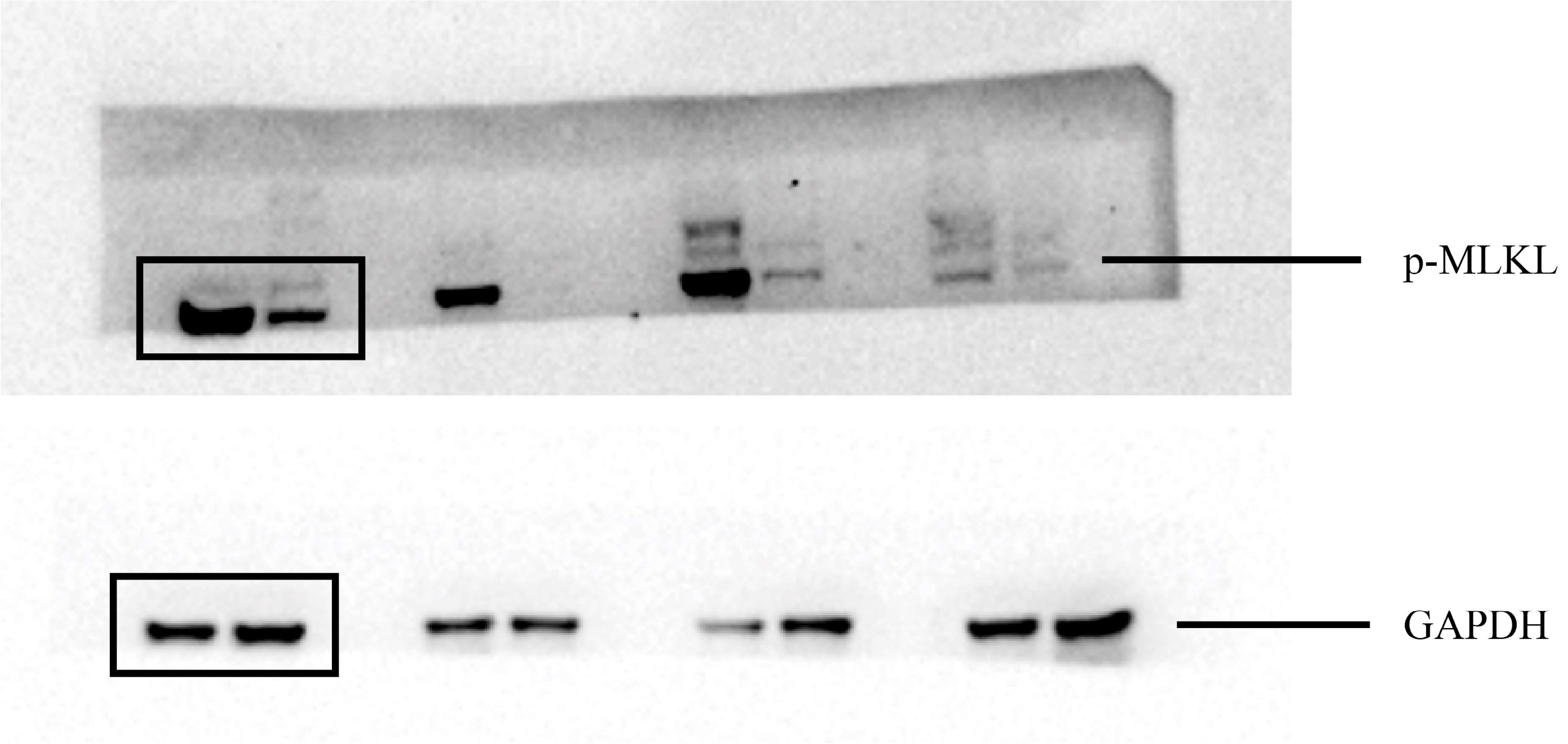

Source data in Figure 4I

A

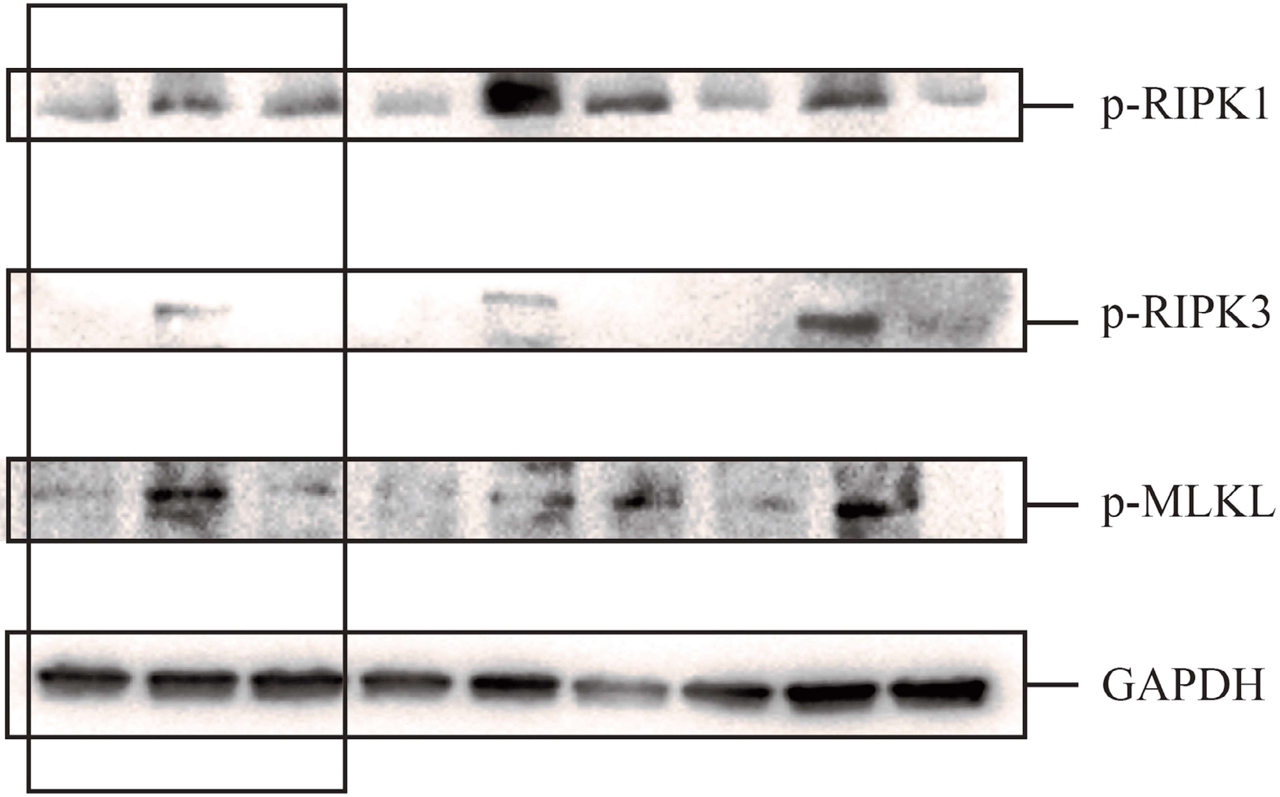

Source data in Figure 5A

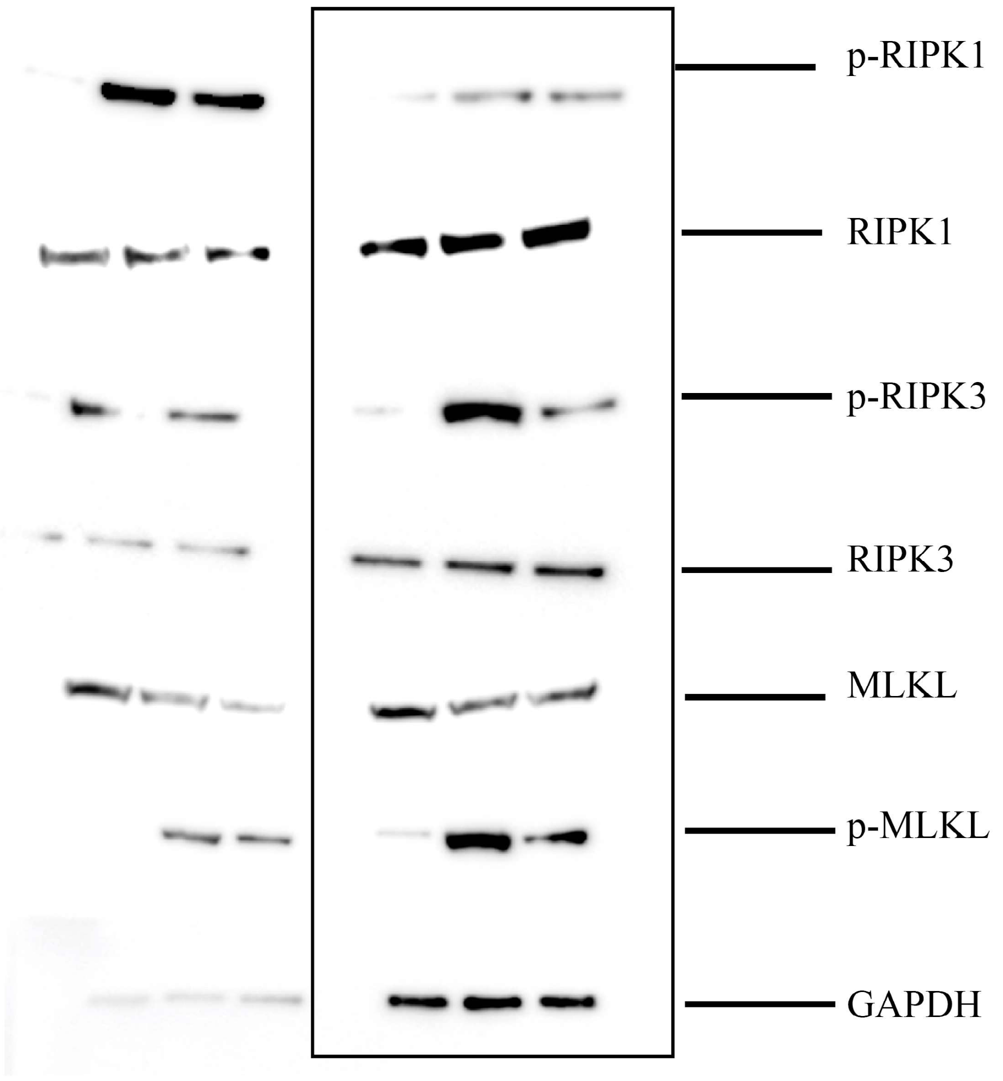


Source data in Figure 5B

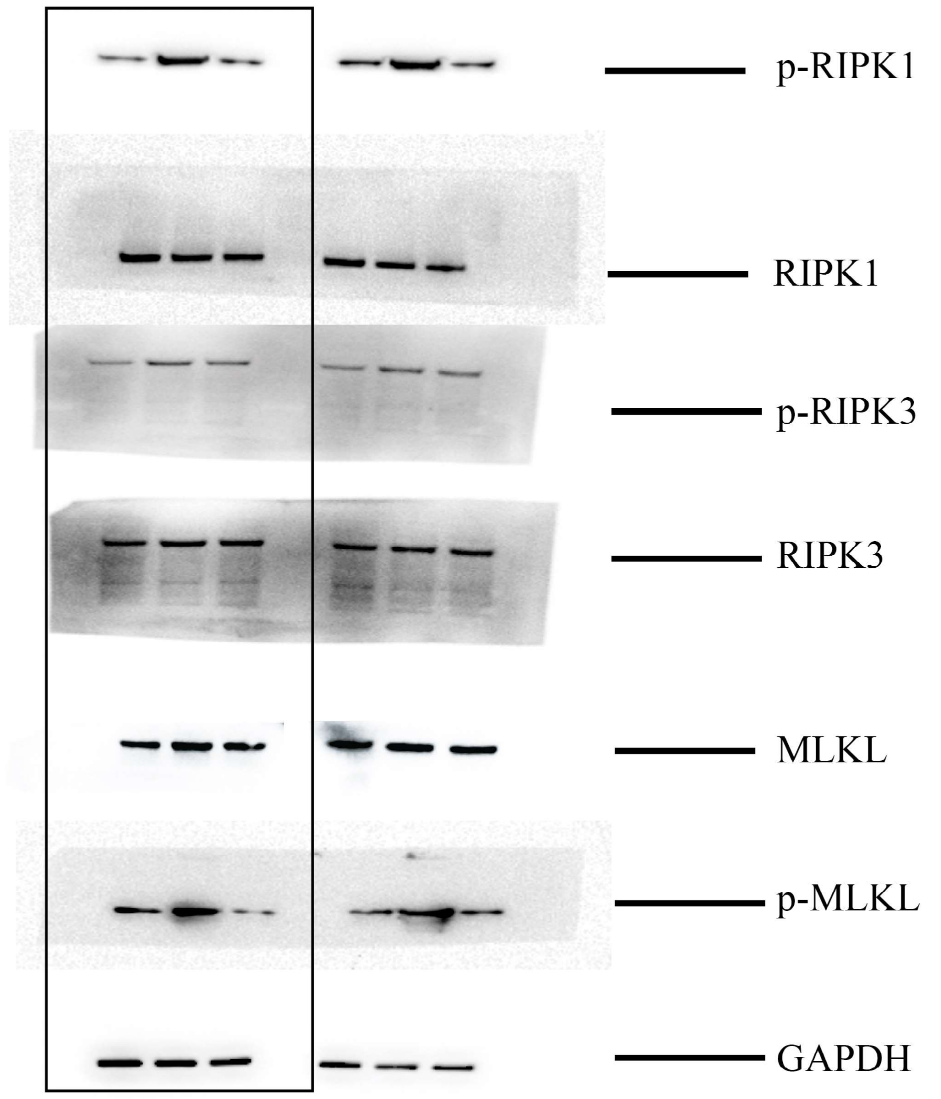


Source data in Figure 5E

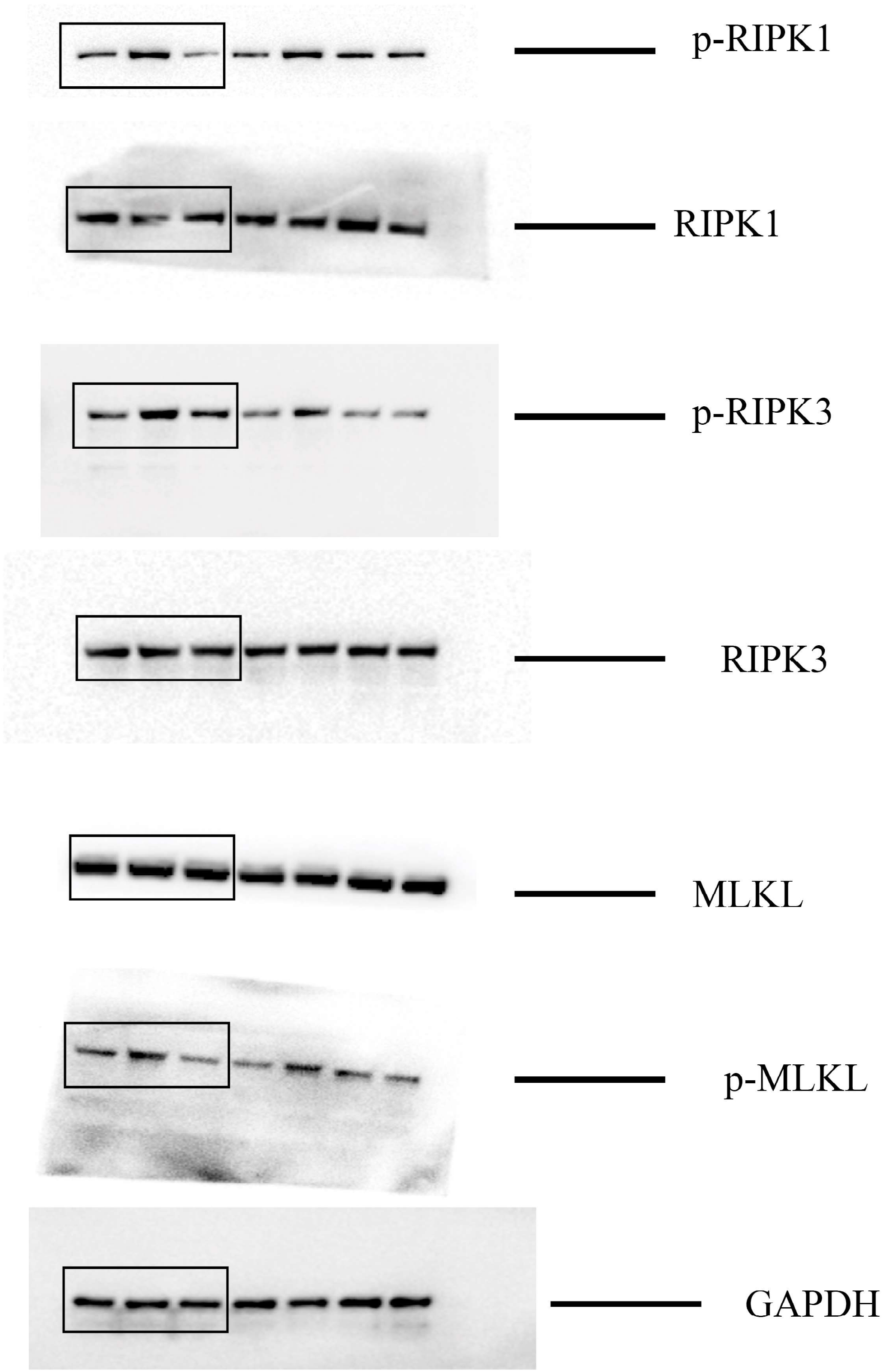


Source data in Figure 5F

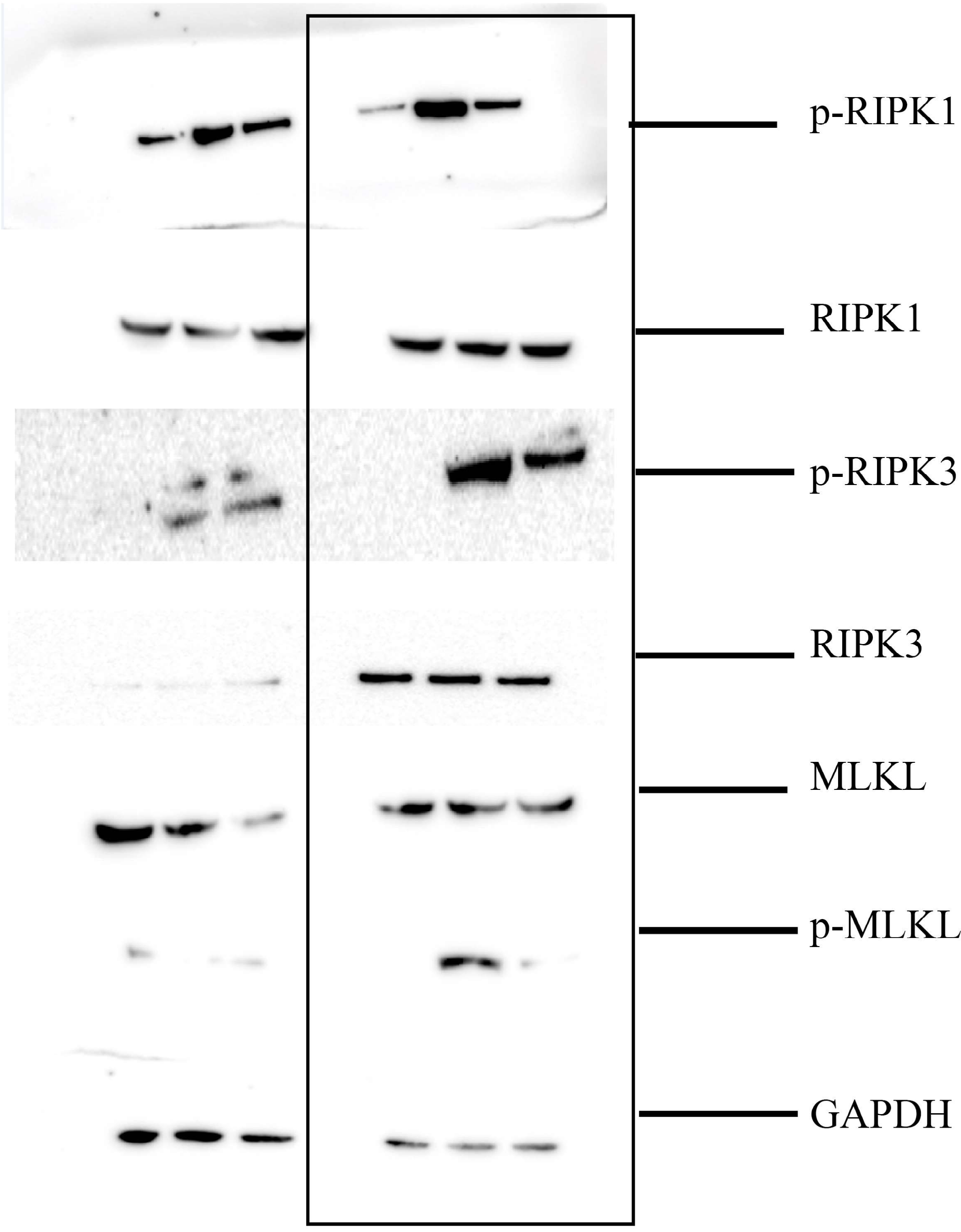

Source data in Figure 6B

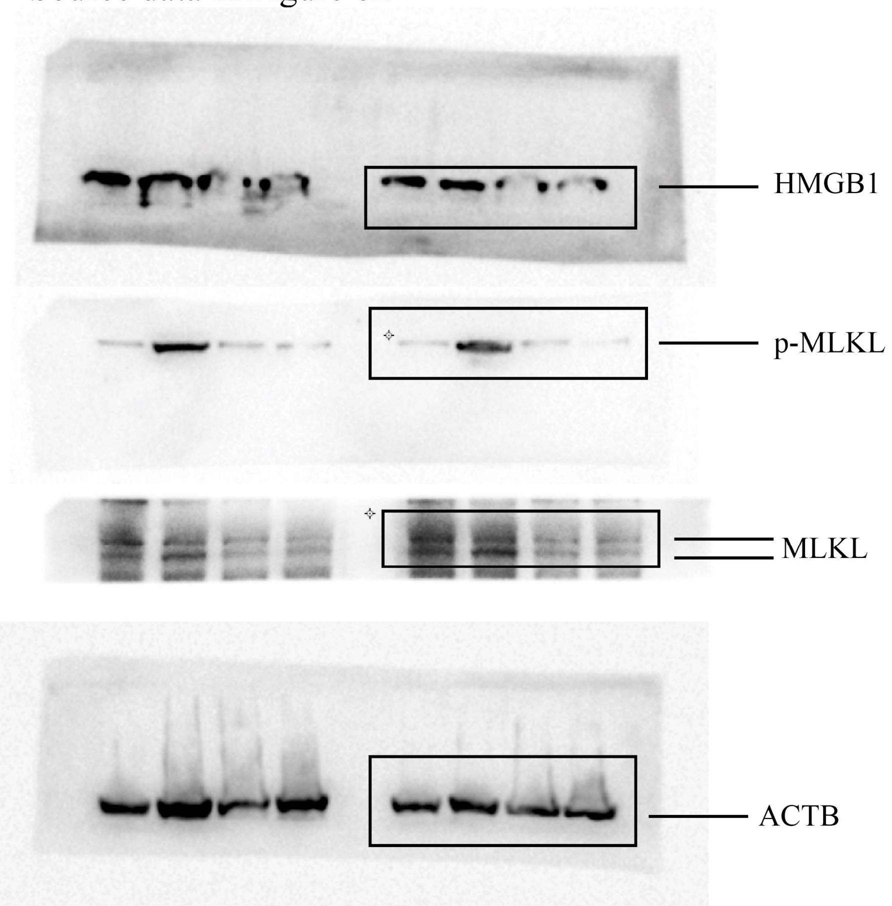


Source data in Figure 6D

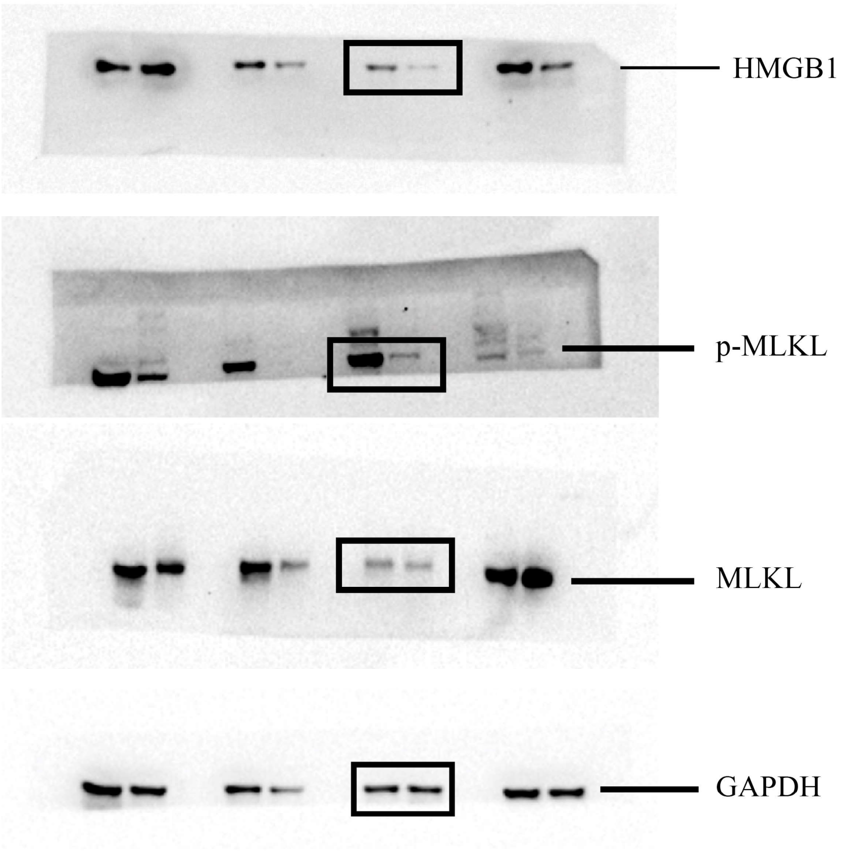


Source data in Figure 6F

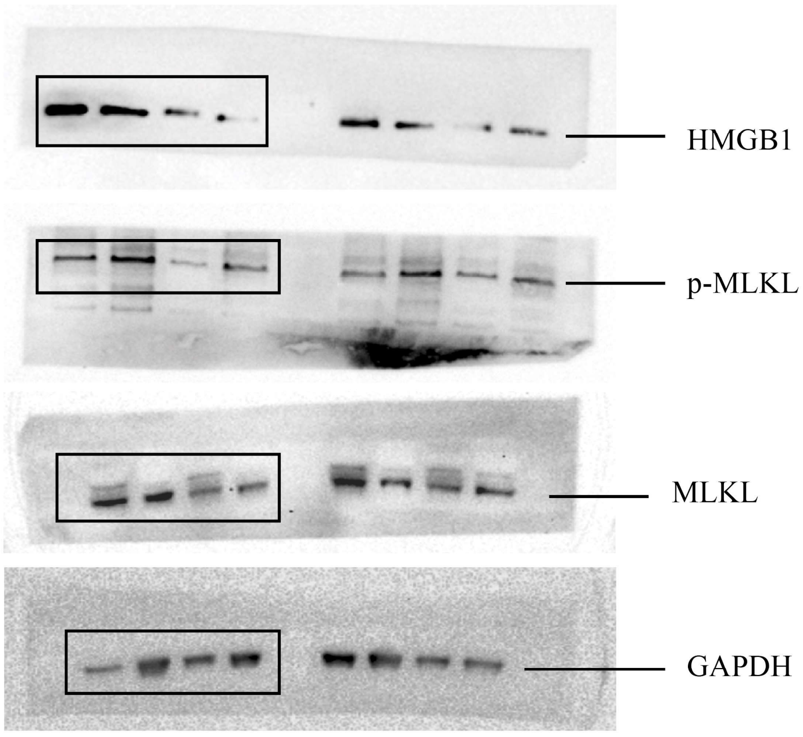


Source data in Figure 6J

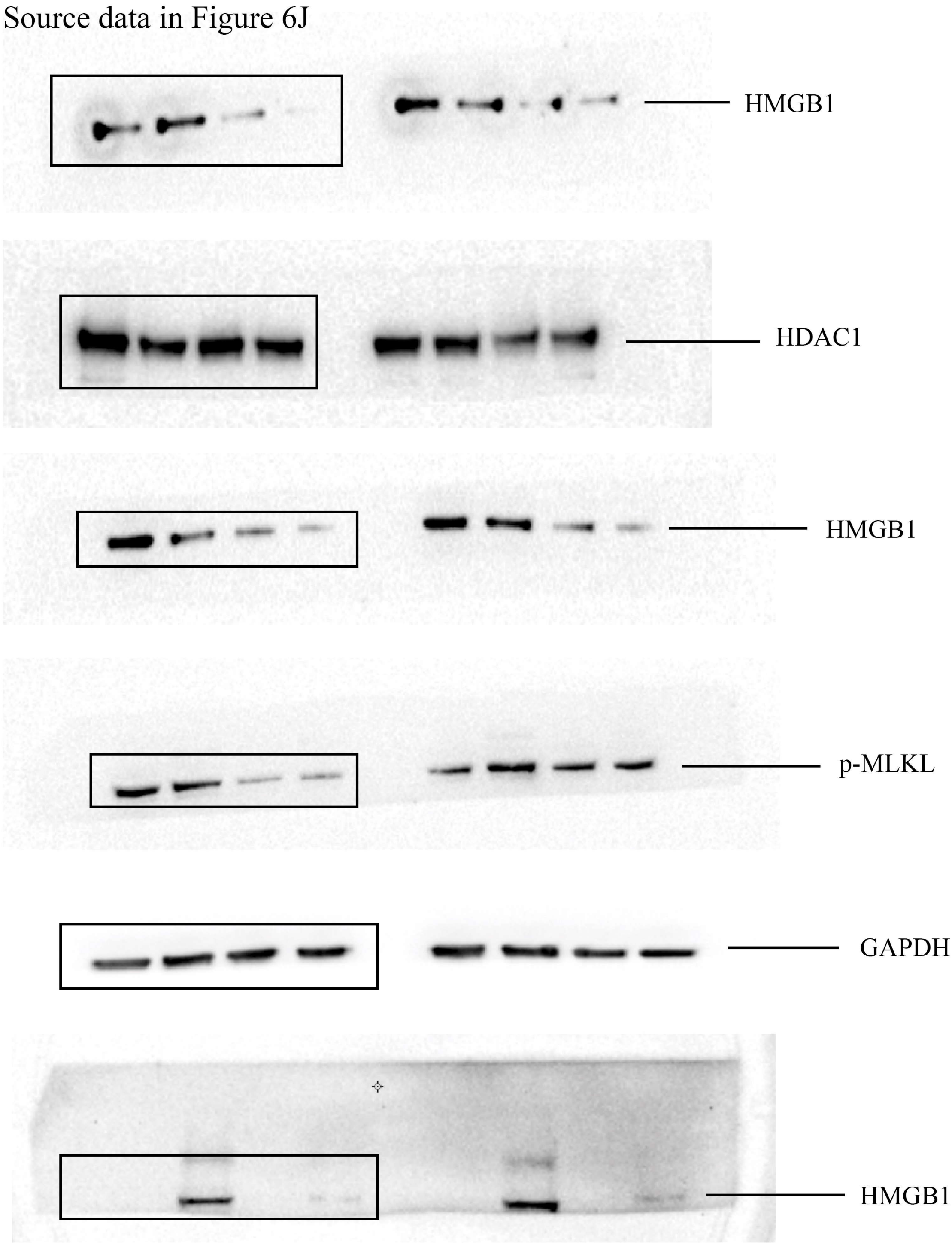

Supplement: Supplementary file 2 — Original Data File [file 41420_2022_1228_MOESM2_ESM.pdf]
